# *Vicia faba* Hypersensitivity and ASA Intolerance in a Farmer: A Case Report

**DOI:** 10.1155/2011/191787

**Published:** 2011-06-08

**Authors:** Elisabetta Damiani, Anna Maria Aloia, Maria Giovanna Priore, Angela Pastore, Stefania Nardulli, Cristina Lippolis, Luigi Macchia, Antonio Ferrannini

**Affiliations:** Division of Allergy and Clinical Immunology, 11, 70124 Bari, Italy

## Abstract

The IgE-mediated allergic reactions to food are caused, generally, by ingestion. However, they can be rarely induced by exposure to airborne food particles through the handling or the cooking. *Vicia faba* is a vegetable which belongs to Legumes or *Fabaceae* family, *Fabales* order. Allergic reactions after ingestion of legumes and cases of asthma after exposure to the cooking vapors have been reported in the literature. A paper assessed the volatile substances (insect repellents) released by *V. faba*. The authors demonstrated that this plant produces several chemical substances, such as small quantities of methyl salicylate. We describe a case of occupational allergy, induced by handling during picking up of fresh broad beans, in a farmer with history of adverse reaction after eating the cooked and raw vegetable.

## 1. Introduction

The IgE-mediated allergic reactions to food are caused, generally, by ingestion. However, they can be rarely induced by exposure to airborne food particles through the handling or the cooking [1]. 

*Vicia faba* is a vegetable which belongs to Legumes or *Fabaceae* family, *Fabales* order. The name “legume or pod” derives from the fruit, which is composed from two symmetrical valves enclosing the seeds [2]. Several legumes were used as food, such as the kidney bean (*Phaseolus vulgaris*), pea (*Pisum sativum*), broad bean (*Vicia faba*), lupin (*Lupinus albus*), chick pea (*Cicer arietinum*), peanut (*Arachis hypogaea*), soy bean (*Glycine max*), and lentil (*Lens culinaris*) [3]. Immunological cross-reactivity has been widely reported in this family but the clinical cross-reactivity is rare [4, 5].

The broad bean is cultivated in Italy, especially in the southern regions and the islands although it is originated in northern Africa and Asia. The plant grows erect and can reach a height of 1.8 m. The leaves are long with jagged edges, and the flowers are white with black stripes and a white and purplish halo with a black spot. The fruit is a green pod with a velvety consistency that gets darker as it matures. Each pod can contain 3–8 oval-shaped seeds that can be consumed both cooked and raw [6].

Allergic reactions, sometimes severe, after ingestion of legumes and cases of asthma after exposure to the cooking vapors have been reported in the literature [7–12].

The presence of 7S (from 10 to 70 kDa) and 11S globulin (from 20–25 to 35–50 kDa) has been demonstrated by Freitas et al. in a study on several legumes, including *V. faba* [13]. In addition, some authors extracted from *V. faba* a VfSBPL (*Vicia faba* sucrose binding-like protein) of 52 kDa that has a 58% homology with the 62 kDa protein contained in soy bean (*Glycine max*) [14].

A paper assessed the volatile substances (insect repellents) released by *V. faba.* The authors demonstrated that this plant produces several chemical substances, such as small quantities of methyl salicylate [15].

A IgE-mediated mechanism has been demonstrated in a series of six patients with referred adverse reactions after eating fresh raw and cooked broad beans and specific IgE to the proteins extracted from the vegetable in two of the six patients' serums. In the remaining four patients, the relationship between history of acetylsalicylic acid (ASA) intolerance and asthmogenic metabolites present in the vegetable has been suggested as a cause of the *V. faba* reactions [16]. 

We describe a case of occupational allergy, induced by handling during picking up of fresh broad beans, in a farmer with history of adverse reaction after eating raw and cooked vegetable.

## 2. Materials and Methods

### 2.1. Patient

A 49-year-old woman, farmer, reported two episodes of adverse reactions induced by raw and boiled broad beans ingestion or handling of fresh vegetable. 

The first episode presented as dyspnea, chest tightness, tachycardia, and malaise after the patient ate fresh raw and boiled broad beans; the second episode presented as a sensation similar to the previous associated with burning eyes and facial oedema while picking up fresh broad beans. On both occasions prompt treatment in an emergency unit relieved the symptoms after approximately two hours. 

In addition, the patient had reported other episodes, related to the picking up of fresh broad beans pods, but in milder form.

The clinical history showed dyspnea and chest tightness after intake of ASA and nonsteroidal anti-inflammatory drugs (NSAIDs). These reactions were diagnosed as adverse drug reactions at another hospital and were prior to those caused by ingestion and handling of fresh broad beans.

The patient picked up the pods of fresh broad beans for 4-5 years, working many hours in large areas used for the cultivation of the vegetable; moreover, she began to show the symptoms during the harvest of the last two years, but after the reaction caused by raw and boiled broad beans ingestion. The respiratory symptoms reported in medical history were absent when she did not work in the picking up of fresh broad beans and when she was at home.

To explain the adverse reactions reported, the patient underwent *in vivo *and *in vitro *tests. In addition, the extracts from fresh cooked and raw broad beans diluted and undiluted were prepared to perform skin prick tests (SPTs) and immunoblotting (IB).

### 2.2. Protein Extraction from Boiled and Raw Fresh Broad Bean

To extract the proteins from the cooked legume, the seeds were boiled for approximately 30 min at 100°C. The extraction was done by homogenization, separately for the boiled and raw seeds, in PBS at 4°C for 24 h. Then the mixtures were centrifuged at 15000 rpm for 1 h at 4°C, and the supernatant was concentrated (Centrifugal Filter Devices, Millipore, Bedford, USA), performing a first centrifugation at 2500 g for 30 min at 4°C and an inverse centrifugation at 2000 g for a further 4 min, again at 4°C. The protein concentration was determined according to Bradford's method [17].

### 2.3. Gel Electrophoresis and Protein Spectrum

For electrophoresis, the extracts were set up in reducing conditions, according to the Laemmli method, adding *β*-mercaptoethanol and heating at 100°C for 5 min [18]. The proteins, contained in the extracts, were separated on a 4–12% polyacrylamide gel (Nupage Bis Tris, Invitrogen, Milan, Italy) for 1 h at 190 V and then stained with colloidal Coomassie blue [19].

### 2.4. Skin Prick Test

The patients underwent SPTs with common inhalant allergens, commercial food extracts including pea, peanut, kidney bean, soy bean (Stallergenes, Milan, Italy), chickpea, and lentil (Lofarma, Milan, Italy), and with extracts of fresh boiled and raw broad beans undiluted and diluted (1 : 1000, 1 : 100, 1 : 10 in saline solution and glycerol) [20]. Saline solution and histamine (10 mg/mL) were used as negative and positive control, respectively.

### 2.5. Immunoblotting

The proteins separated on polyacrylamide gel were transferred onto a nitrocellulose membrane (Schleicher & Schuell, Biosciences, Germany) [21]. The membranes, after saturation, were incubated at 4°C overnight with the patient's serum. After several washes, the membranes were incubated with human anti-IgE conjugated with peroxidase for 2 h. Bound specific IgE was detected by chemiluminescence.

## 3. Results

### 3.1. Protein Extracts and Spectrum

The protein concentration from the raw and boiled seeds of *V. faba* extracts was 5.48 mg/mL and 4.43 mg/mL, respectively. Molecular analysis showed the presence of proteins weighing from 12, 16, 20, 32, 45 to 60–65 kDa (Quantity One Basic, BioRad, Milan, Italy).

### 3.2. Skin Prick Test

Prick tests with common inhalant allergens and commercial food extracts were negative. SPTs performed with extracts of fresh raw and boiled broad beans undiluted and diluted were stopped at raw broad beans extract diluted 1 : 1000, because the patient had positive wheal and symptoms, such as tachycardia, malaise, and hypotension. This reaction required prompt pharmacological therapy with remission of the symptoms.

SPTs performed with undiluted and diluted extracts of raw and boiled fresh broad beans in controls were negative.

### 3.3. Immunoblotting

The IB performed with the patient's serum showed specific IgE reactivity to the 60–65 kDa protein.

## 4. Discussion and Conclusion

Legumes are vegetables that are responsible for allergic reactions especially after ingestion, but they occasionally can induce occupational allergies, as in the case described by Bush and Cohen about asthma induced by exposure to soybean dust [22].

Our patient experienced dyspnea, chest tightness, tachycardia, and malaise both after ingestion and after handling of fresh broad beans while picking up. These clinical manifestations recurred during the performance of SPTs with extracts of fresh raw broad beans diluted to a low concentration (1 : 1000) that required discontinuation testing and pharmacological therapy. Daroca et al. reported cases of respiratory symptoms appeared after handling of green beans or green beans and chards together in patients who ingested the same food without reactions. The IB performed with patient's serum showed several differences, although minimal, about the reactivity of IgE between IB with the raw extract and the blotting with boiled extract. This may be related to the lability of allergens of green beans to cooking, which would explain the tolerance to the ingestion of cooked foods [23]. Another case of asthma induced by handling and cooking vapors of green beans has been described by Igea et al. in a housewife who tolerates ingestion of vegetable. In this case, the presence of protein heat-labile in vegetable has been suggested [24]. In our case, the patient did not tolerate the ingestion of the native (raw) and boiled food and the proteins are well preserved also in the cooked extract.

The IB shows IgE reactive against the protein of molecular weight of 60–65 kDa, present in the fresh raw and boiled broad beans extract. This demonstrates that the reaction experienced by the patient can be IgE mediated.

The presence of salicylates in vegetable and the role of salicylic acid as defense response to pathogens are known in the literature [25–27]. In addition, after external stress stimuli, the *V. faba* produces small quantities of methyl salicylate and others substance that can be measured in the surrounding air [15]. A series of patients with ASA intolerance and reactions after ingestion of *V. faba* cooked and raw seeds had assumed to the authors a asthmogenic role of some metabolites of methyl salicylate present in the plant [16]. In several cases reported in the literature, the reactions caused by the inhalation of food allergens appear, generally, in domestic environment [7, 8, 23, 24] or in industrial environment [22].

Our patient reported history of adverse reactions after intake of NSAIDs and ASA, prior to those caused by ingestion and handling of fresh broad beans. Moreover, the reactions that the patient experienced during working, picking up the pods of *V. faba* (without the concomitant ingestion of seeds), were at first slight and after successive exposures had become more serious. The evolution of symptoms has suspected that the patient's ASA intolerance could have had a significant role. In fact, the methyl salicylate, released from the vegetable in the surrounding air and inhaled by the patient during the picking up of the pods, may have induced the respiratory symptoms due to previous sensitization to the ASA. After the investigations performed, by us the patient avoided her carrying out her work where *V. faba* was cultivated.

Furthermore, we have demonstrated an IgE-mediated mechanism of the reactions after ingestion of the seeds of broad beans, but we cannot exclude that the reactions during the picking up of broad beans could be caused by the inhalation of airborne allergens. 

We concluded that the reactions after ingestion of fresh broad beans are IgE mediated, while the reactions that occurred during the harvest of vegetable can be explained by two diagnostic hypotheses:

1. exposure to airborne allergens responsible for an immediate response,
2. exposure to airborne salicylates responsible for a intolerance reaction.

Future studies could confirm these hypotheses and clarify this phenomenon. 

We cannot exclude, however, that reports may increase over time, because the plants and the vegetables are increasingly exposed to aggressive agents and then tend to respond to these stimuli by increasing the synthesis of allergenic defensive proteins and volatile substances. This situation, therefore, could lead to an increase of cases of occupational allergies induced by volatile substances released from the vegetables in the surrounding air.

##  Conflict of Interests

The authors declare that they have no financial relationship with any biotechnology manufacturers that has an interest in the subject matter or materials discussed in the submitted paper.
